# Concerning Tooth Extracting

**Published:** 1861-08

**Authors:** J. A. M’Clelland


### CONCERNING TOOTH EXTRACTING.

BY J. A. M'CLELLAND.

(Read before the Kentucky State Dental Association, at Louisville, April 9th, 1861.)

NOTWITHSTANDING the advanced state of dental science, this, the *primitive* branch of our profession, continues to be one of the most common operations the dentist is called upon to perform.

The cause of this great sacrifice of organs so important to health, comfort, correct enunciation, and appearance, is not so much due to inadequacy of our art to save them, as to a general lack of knowledge in reference to the teeth, the importance of their preservation, and the treatment they should receive.

The early decay and premature loss of the teeth conduce not a little to the physical degeneracy of our race, and yet the unnecessary sacrifice of these organs, witnessed daily, is truly painful to the philanthropic dentist.

As a remedy, the dissemination of dental knowledge among the people would be most effectual. They do not understand the first law of nature (self-preservation), in its application to the teeth. They need *waking up* to the important truth, that *cleanliness* and *good dental operations* are the *salvation* of these organs. The question naturally arises, by whom and how shall this missionary work be performed ?

The operative dentist sees daily how little is known about the teeth, even by parents, when he awakens their surprise by telling them that the tooth they wish extracted for their child is not a deciduous tooth, as they supposed, but one of the second set, and probably that one or two other new teeth require filling. Cases like these afford the dentist an opportunity to impart a useful lesson, which humanity and the good of his profession demands that he should do. A dentist only does his *duty* when he indicates to his patient that certain teeth need filling or other treatment. His motives may sometimes be impeached, but he will be remembered more kindly when the truth of his advice is *felt*, though it should come *painfully* too late. That class of patients who have so much of the doubting Thomas in their nature, that when told their teeth need filling, will not believe until they miss the bread and butter that hides away in the mammoth cavities, do not merit the sympathy of the profession, unless it be for their great *scepticism*, which may bring them to need their *grinders* in the *future*.

It is unnecessary to attempt to enumerate the causes that lead to the extraction of so many teeth. It is not, however, always owing to neglect on the part of the patient. Inferior dental operations have a large share of sin to answer. As where teeth have been imperfectly filled, or have been embraced by clasps for the support of artificial teeth, when an atmospheric pressure plate could have been worn quite as well.

The practice of extracting teeth, in view of supplying artificial substitutes, in cases where the natural teeth could have been filled, and for years rendered more serviceable than the artificial ones, is by far too common. If we pride ourselves on supplying inimitable *imitations* of nature's pearly arches, we should pride ourselves still *more* in being able to preserve the *natural* pearls of price.

Teeth must sometimes be extracted, and to perform this operation well is no less an accomplishment in the dental art, than is good execution of instrumental music an accomplishment in the art of music—both requiring, in addition to correct theoretical knowledge, much practice in the use of the instrument. It is not enough to know how to shave and champoo, or simply apply and turn a key with clenched fist and eyes shut, regardless of consequences. Such operations are generally barbarous, not to say hazardous in the extreme.

If there is one operation more than another that the dentist should go about understandingly, it is that of extracting teeth. Although the operation is generally accomplished without difficulty, cases occasionally present themselves, which tax the skill and ingenuity of the experienced and scientific operator. If we have a tooth to fill—even the most difficult cavity, or a full artificial denture to insert, we can predict with almost unerring certainty what degree of success will attend our efforts; but in the extraction of a tooth, oftener perhaps than in any other operation, are we liable to be disappointed in our expectations. We can not always calculate the strength of the tooth or the resistance to be overcome, and the latter often proves greater than the former.

So varied are the cases as to age, temperament and constitution of the patient, quality and condition of the teeth and osseous system, that to diagnose and prognose correctly, a wide range of anatomical, physiological, pathological, and *experimental* knowledge are brought into requisition. Without such knowledge, the operator must grope his way in the dark, committing many blunders, and by his frequent failures to accomplish what he perhaps so flatteringly promised, receives what he merits, the disapprobation of his patient and the unenviable reputation of a bungler.

A correct diagnosis is the key to success—not less in our profession, than in the practice of general medicine. A physician may err in diagnosis, and fail of success in a case; his blunders are buried with his patient, and his reputation sustains no loss. Not so with the dentist; he must *demonstrate* everything; perfection is demanded in every operation. His successes are received as a matter of course, while if he fail in one case, his skill is questioned. We should, therefore, examine well the case before expressing an opinion. If it be in reference to the extraction of a tooth, and we entertain any doubt of success attending our first effort, it is better to tell the patient the state of the case, that they may be prepared to do their part, and afford the operator every facility they can for success. By this course, in difficult cases, the confidence of the patient will be retained, and even strengthened to perseverance, in prolonged operations. It is the practice of some, however, to pursue an opposite course under all circumstances, flattering their patients with success and no pain, when it too frequently results all pain and no success.

There is a vast difference in patients as to their fortitude to bear pain. While a few have the will to submit and the nerve to bear the operation of extraction with apparent indifference ; to the mind of the nervous and timid, nothing is more terrifying than the idea of having a tooth forcibly removed from the socket where it grew. Patients of the latter class require *sympathy*, and it is the duty of the dentist to give them all the encouragement and assurance he can consistently. When we tell them, as we are apt to do, that "it will not hurt more than they can well bear, and just summons a little courage, and the operation will soon be over," if they do not readily submit, we should not lose our temper, dash down the instrument, and ask them gruffly what they came here for ? Timid patients may be thus frightened into submission, but their fear and abhorrence of dental operations are confirmed for life. Firmness with patients is sometimes necessary ; but we should ever be guided by our judgment what course to pursue.

When a patient consumes an unnecessary amount of time, and taxes our patience, we may charge them accordingly, and they will see the justice of the charge. It is only right to charge a fee for time unnecessarily consumed, even if the patient does not have the tooth extracted. The physician charges for advice, whether the patient takes the prescription or not, and the services of the dentist who is thoroughly qualified in his profession are not less valuable than those of the physician. The charges, however, as well as the treatment, must be varied to suit the case. The dignity of our profession is not lowered by *charitable* operations.

That class of patients who are most likely to consume the time of the dentist unnecessarily generally lack fortitude, or in other words, have not the force of *will* to bring their courage up to the extracting point. A little brandy, administered in such cases, will generally have a beneficial effect.

In debilitated constitutions, and in those which, from idiosyncracy, are liable to syncope from slight causes, brandy should be administered before extracting teeth, and in no case where this stimulus is properly administered, need there be any apprehension of syncope.

A lady patient said that she *always* fainted when she had a tooth extracted (and she had lost six or seven), and was accordingly making preparations for the event, remarking that she would soon revive, and not to be alarmed. Brandy was given, and there was no indication of syncope. Patients of this peculiar idiosyncracy of constitution will bear double the potion of brandy when a tooth is to be extracted, that they would under ordinary circumstances.

In temperaments where the nervous highly predominates, with excitability, and particularly if the patient be a female, alcoholic stimulus should be withheld, as it would tend greatly to increase excitability, and leave the patient with an intense headache. To patients of this temperament a cup of strong tea or coffee would be a good substitute for the brandy.

It is a matter of some importance to know when and under what circumstances teeth should be extracted. Experience has sufficiently proven that teeth may be preserved and rendered useful for many years after they have decayed to the nerve. Hence, it is not always necessary nor advisable to extract teeth simply because they ache, and it may not be proper to extract teeth at all times, when aching, although they may be regarded worthless.

The operation of extraction is most painful when the tooth is affected with periostitis, the pain varying in degree in proportion as the inflammation is advanced, and yet this is the very time when patients are most apt to apply to have *dead* teeth and fangs removed. Notwithstanding the soreness, as a rule, it is better to extract at once than to relieve the case, even if it could be done, as patients are apt to delay their removal when the inflammation and soreness subside.

Under some circumstances, however, it is better to relieve the case, if possible, without extracting, until the patient is better able to bear the operation. This may often be done in the early stage by opening up the nerve canal—giving vent to the mephitic gas in the fang, and fluid that may have accumulated at the apex of the fang. Should this fail, leeching and a cooling laxative will generally have the desired effect.

In cases where periostitis exists, the pain often continues unabated for some time after the tooth is extracted, and in some instances for many hours, leading the patient to suppose that the operation was not skillfully performed. In view of this, and as the dentist never receives in such cases the credit of "not hurting a bit," it is well to prepare the patient's mind for what may follow. In these and similar cases, prompt relief may generally be given by application to the gum of a little cotton, slightly moistened with chloroform containing gum camphor, and covered with tin foil to retain the vapor.

There are other conditions under which it may not always be proper to extract teeth, as in cases of pregnant females—(though it is not expected that the dentist can always know when his patient is in this condition.) Physicians generally object to the practice, regarding it as unsafe. Toothaches at those times they usually attribute to the condition of the system, regarding them as merely sympathetic. Experienced dental practitioners, however, know the liability of physicians to err in their diagnosis of odontalgia. If the case were clearly idiopathic, it would, in all probability, be pronounced by them sympathetic.

Females are, doubtless, more subject to toothache during the period of gestation. Owing to the activity of life principle, the nervous system is highly susceptible to impressions, and slight causes produce irritation. Long continued suffering from toothache and loss of sleep at such a time would be far more injurious than the slight and temporary shock of having the tooth extracted; hence, we seldom hesitate to extract teeth, knowing the patient to be enciente, unless she is of an *extremely nervous* and *excitable* temperament, and very much *fears* the operation. In this case, it would be advisable to adopt palliative treatment.

Mrs. T—, whose teeth and gums were in a dreadfully diseased condition, caused her much suffering and ill health. She had borne four or five children, and was pregnant when she applied to have her teeth extracted. This, however, was her own secret at the time. She subsequently made known the fact, and stated that during the whole of each previous term of pregnancy, she suffered constantly with her teeth. This time she resolved to have them taken out. They were extracted at two sittings. She was four months advanced when four teeth were taken out. In three weeks after, six others, mostly molars, were extracted. No unpleasant effects followed, except temporary sickness from the effects of the brandy she had taken. She experienced no further trouble with her gums or remaining teeth, and her health rapidly improved. Her complexion, which before was pale and sallow, with puffiness about the face, became fresh and rosy. Her child was a fine boy, and *free from any marks.*

